# Exosome-derived small non-coding RNAs reveal immune response upon grafting transplantation in *Pinctada fucata* (Mollusca)

**DOI:** 10.1098/rsob.210317

**Published:** 2022-05-04

**Authors:** Songqian Huang, Shinya Nishiumi, Md Asaduzzaman, Yida Pan, Guanting Liu, Kazutoshi Yoshitake, Kaoru Maeyama, Shigeharu Kinoshita, Kiyohito Nagai, Shugo Watabe, Tetsuhiko Yoshida, Shuichi Asakawa

**Affiliations:** ^1^ Department of Aquatic Bioscience, Graduate School of Agricultural and Life Science, The University of Tokyo, Tokyo 113-8657, Japan; ^2^ Department of Marine Bioresources Science, Faculty of Fisheries, Chittagong Veterinary and Animal Sciences University, Khulshi 4225, Chittagong, Bangladesh; ^3^ Mikimoto Pharmaceutical Co., Ltd., Kurose 1425, Ise, Mie 516-8581, Japan; ^4^ Pearl Research Laboratory, K. Mikimoto & Co., Ltd., Osaki Hazako 923, Hamajima, Shima, Mie 517-0403, Japan; ^5^ School of Marine Biosciences, Kitasato University, Minami-ku, Sagamihara, Kanagawa 252-0313, Japan; ^6^ Institute for Advanced Sciences, Toagosei Co., Ltd., Tsukuba, Ibaraki 300-2611, Japan

**Keywords:** exosome, small non-coding RNAs, miRNA, piRNA, immune response, *Pinctada fucata*

## Abstract

Exosomes, a subset of small extracellular vesicles, carry various nucleic acids, proteins, lipids, amino acids and metabolites. They function as a mode of intercellular communication and molecular transfer. Exosome cargo molecules, including small non-coding RNAs (sncRNAs), are involved in the immune response in various organisms. However, the role of exosome-derived sncRNAs in immune responses in molluscs remains unclear. Here, we aimed to reveal the sncRNAs involved in the immune response during grafting transplantation by the pearl oyster *Pinctada fucata*. Exosomes were successfully extracted from the *P. fucata* haemolymph during graft transplantation. Abundant microRNAs (miRNAs) and PIWI-interacting RNAs (piRNAs) were simultaneously discovered in *P. fucata* exosomes by small RNA sequencing. The expression patterns of the miRNAs and piRNAs at the grafting and initial stages were not substantially different, but varied significantly between the initial and later stages. Target prediction and functional analysis indicate that these miRNAs and piRNAs are related to immune response upon grafting transplantation, whereas piRNAs may also be associated with transposon silencing by targeting with genome transposon elements. This work provides the basis for a functional understanding of exosome-derived sncRNAs and helps to gain further insight into the PIWI/piRNA pathway function outside of germline cells in molluscs.

## Graphical abstract

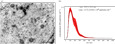

## 1. Introduction

Cells from all domains of life secrete extracellular vesicles (EVs), which act as intercellular communication tools and contain functional nucleic acids, proteins, lipids, amino acids and metabolites [1]. Exosomes, a class of EVs with a diameter of approximately 30–150 nm, are produced as components of multivesicular bodies (MVBs). Exosomes are released from cells when MVBs fuse with the cell surface, and are transferred and taken up by recipient cells to exchange internal components [2]. Exosomes are of particular interest in biology because of their ubiquitous detection in biological fluids and complex constituents associated with immune responses, ageing, organ homeostasis, viral pathogenicity, pregnancy and diseases [3–5]. Studies relevant to exosomes have largely focused on model animals or higher organisms involved in immune response, health and disease [6,7]. With the development of biotechnology and the continuous expansion of research scope, exosomes have been detected in various non-model animal body fluids, including arthropods [8], crustaceans [9], molluscs [10–12] and teleosts [13,14].

MicroRNAs (miRNAs) are small non-coding RNA (sncRNA) molecules that are 20–23 nucleotides (nt) in length. They post-transcriptionally regulate the expression of target genes by binding to the 3′-untranslated regions (3′UTRs) of mRNA, which are involved in cell proliferation, tissue differentiation, embryonic development, metabolism and apoptosis [15,16]. PIWI-interacting RNAs (piRNAs) comprise a class of sncRNA molecules that are 24–31 nt in length. miRNAs are processed from long, single-stranded transcripts to form a short hairpin structure associated with AGO subfamily members of the Argonaute proteins [17], whereas piRNAs are generated from long, single-stranded precursors associated with PIWI subfamily members [18]. piRNA biogenesis is classified into two categories, namely the primary and secondary biogenesis pathways. In the primary biogenesis pathway, long piRNA precursors are transcribed from specific genomic loci called piRNA clusters, cleaved and modified by complicated biogenesis factors in the cytoplasm [19,20]. Subsequently, primary piRNAs are subjected to an amplification system to enforce the high expression of piRNAs, which is called the amplification loop or the ping-pong cycle [21].

Cells secrete various EVs, including exosomes, into the internal environment for various forms of cell-to-cell communication [22]. Exosomes act as a natural material transport capsule for intercellular communication to realize the gene expression regulatory activity (RNAi) of sncRNAs [23,24]. miRNA-containing exosomes have been well studied in various animals and humans [25]. However, a few studies have also indicated that piRNAs are detected in exosomes and play key roles in disease and sex development [26–28]. In molluscs, shell and pearl formation is a complex biomineralization process that involves extensive participation of cells and secreted exosomes [28]. The epidermis, gills and feet of the mantle absorb Ca^2+^ and ${\mathrm{HCO}}_{3}^{-}$, which are transported to the mantle by haemocytes and exosomes and secreted by outer mantle epithelial (OME) cells. The shell matrix proteins (SMPs), mostly secreted from OMEs, are directly delivered to the mineralization site via either exosomes or the classical secretory pathway [29,30]. To date, abundant SMPs, which play a vital role in shell and pearl formation, have been identified [31–33]. However, the process by which pearl formation occurs remains poorly understood.

Currently, most studies on exosomes are primarily focused on their roles in disease and immune response [34,35], while exosomes are also involved in shell and pearl formation in molluscs [28]. In pearl farming, small pieces of mantle tissue from donor oysters are implanted into the gonad of host oysters along with an inorganic bead (termed the ‘nucleus’) for large-scale production of artificial pearls [33,36]. Surgical implantation of the mantle can induce an immune reaction in response to transplantation for the survival of the host oyster [37]. Therefore, a vast number of immune-related genes are activated upon transplantation [33,38–40]. However, the exosome-mediated immunological reaction that occurs in host oysters during the subsequent stages of pearl sac formation is still unclear. Accordingly, an improved understanding of exosome-mediated immune responses in host oysters upon acceptance of a transplant is required to further improve the effectiveness of pearl production. In this study, we aimed to reveal the function of exosome-derived sncRNAs upon mantle grafting using small RNA sequencing (sRNA-seq) profiling. Intriguingly, both miRNAs and piRNAs were discovered in the exosomes of *Pinctada fucata*, and their target prediction and functional analysis indicated that these small RNAs are related to the immune response upon grafting transplantation.

## 2. Results

### 2.1. Identification and characterization of exosomes

**Figure 1.**
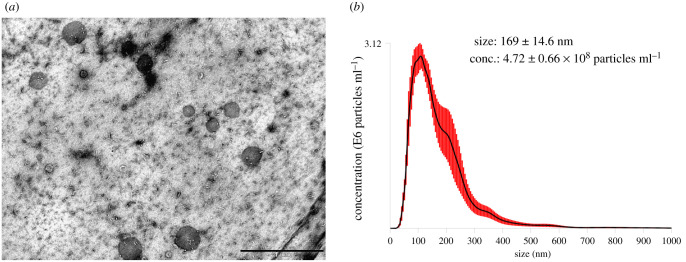
The exosomes isolated from *Pinctada fucata* were detected by transmission electron microscopy (*a*) and nanosight particle tracking analysis (*b*). Scale bar = 1 µm.

Mantle pieces were sheared from donor oysters and implanted into the gonads of host oysters along with an inorganic bead for pearl culture. The haemolymph was collected from host oysters during grafting transplantation for exosome isolation and purification. Exosome morphologies were detected by transmission electron microscopy (TEM) and size by nanoparticle tracking analysis. As shown in figure 1*a*, typical spherical structures of 50–200 nm were observed in the purified exosome preparation. Nanoparticle tracking analysis indicated that the concentration of exosomes was 4.72 ± 0.66 × 10^8^ particles ml^−1^ with an average diameter of 169 ± 14.6 nm (figure 1*b*). All the measurement results suggested that the exosome preparation isolated from *P. fucata* haemolymph contains a heterogeneous mixture of exosomes and macrovesicles.

### 2.2. Small RNA sequencing of exosomes

**Figure 2.**
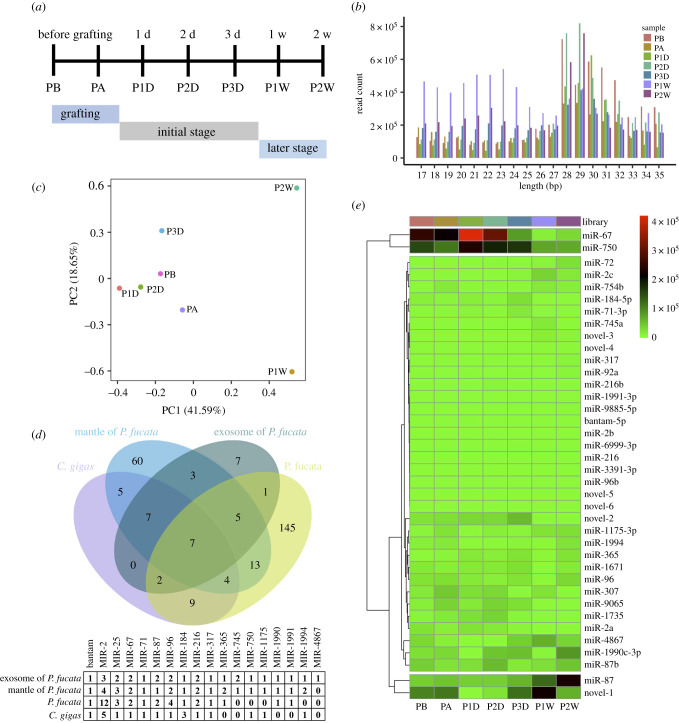
Small RNAs identified in *Pinctada fucata* exosome. (*a*) Experimental design for mantle grafting and sampling schedule; (*b*) length distribution of clean reads by small RNA sequencing during mantle grafting; (*c*) principal component analysis of miRNA expression patterns in *P. fucata* exosome during mantle grafting; (*d*) Venn diagram of the overlap of known miRNA profiles and conserved miRNA family in *P. fucata* and related species. Thirty-two known miRNAs were identified from *P. fucata* exosomes in the present study, 186 known miRNAs from multiple somatic and gonadal tissues of *P. fucata* [41], 104 known miRNAs from mantle tissues of *P. fucata martensii* using the same analysis pipeline [42] and 54 known miRNAs from *Crassostrea gigas* deposited in MirGeneDB 2.1 [43]. (*e*) Heatmap of identified miRNA expression patterns in all investigated samples of *P. fucata*. PA, just after grafting; PB, before grafting; P1D, 1 day post-grafting; P2D, 2 days post-grafting; P3D, 3 days post-grafting; P1W, 1 week post-grafting; P2W, 2 weeks post-grafting.

Total RNA extracted from exosomes was used to construct libraries for sRNA-seq upon grafting transplantation (figure 2*a*). A total of 84.87 million raw reads were harvested from 21 libraries (electronic supplementary material, table S1). After removing the low-quality reads, adapter sequences and those with sizes outside the 15–35 nt range, a total of 39.99 million clean reads remained for statistical analysis of read length (figure 2*b*). Except for the miRNA-sized (20–24 nt) reads detected, predominant piRNA-sized reads (25–31 nt) were also discovered in the sRNA-seq of *P. fucata* exosomes, except for predominant miRNA-sized reads in P1W. After removing genome unmapped reads and known RNAs in Rfam, 37.24 million reads (43.88% of raw reads) remained for miRNA and piRNA identification and characterization.

### 2.3. MicroRNA identification in *P. fucata* exosomes

A total of 32 known and six novel miRNAs were identified, with a large variation in expression levels (electronic supplementary material, table S2). The miRNA counts were normalized according to the reads per million reads (RPM) method. The principal component analysis of miRNA expression showed that the miRNA expression patterns at the grafting and initial stages were not substantially different, but varied significantly between the initial and later stages (figure 2*c*). Among these miRNAs, seven of the 32 known miRNAs, namely miR-2a/b/c, miR-71, miR-317, miR-184 and miR-1990c, were simultaneously discovered in the exosomes and related species, while seven miRNAs, namely miR-745b, miR-1735, miR-3391, miR-4867, miR-6999, miR-9065 and miR-9885, were specifically expressed in *P. fucata* exosomes (figure 2*d*). Several immune-related miRNAs were observed in *P. fucata* exosomes, including miR-2a/b/c and miR-92. Meanwhile, miRNAs, namely miR-750, miR-1990c, miR-1991 and miR-1994, which were especially observed in molluscs, were also detected. Among these miRNAs, miR-67 and miR-750 were upregulated at the initial stage of mantle grafting (P1D, P2D and P3D), whereas miR-87 and novel-1 were downregulated at later stages (P1W and P2W) (figure 2*e*).

### 2.4. PIWI-interacting RNA processing in *P. fucata* exosomes

**Figure 3.**
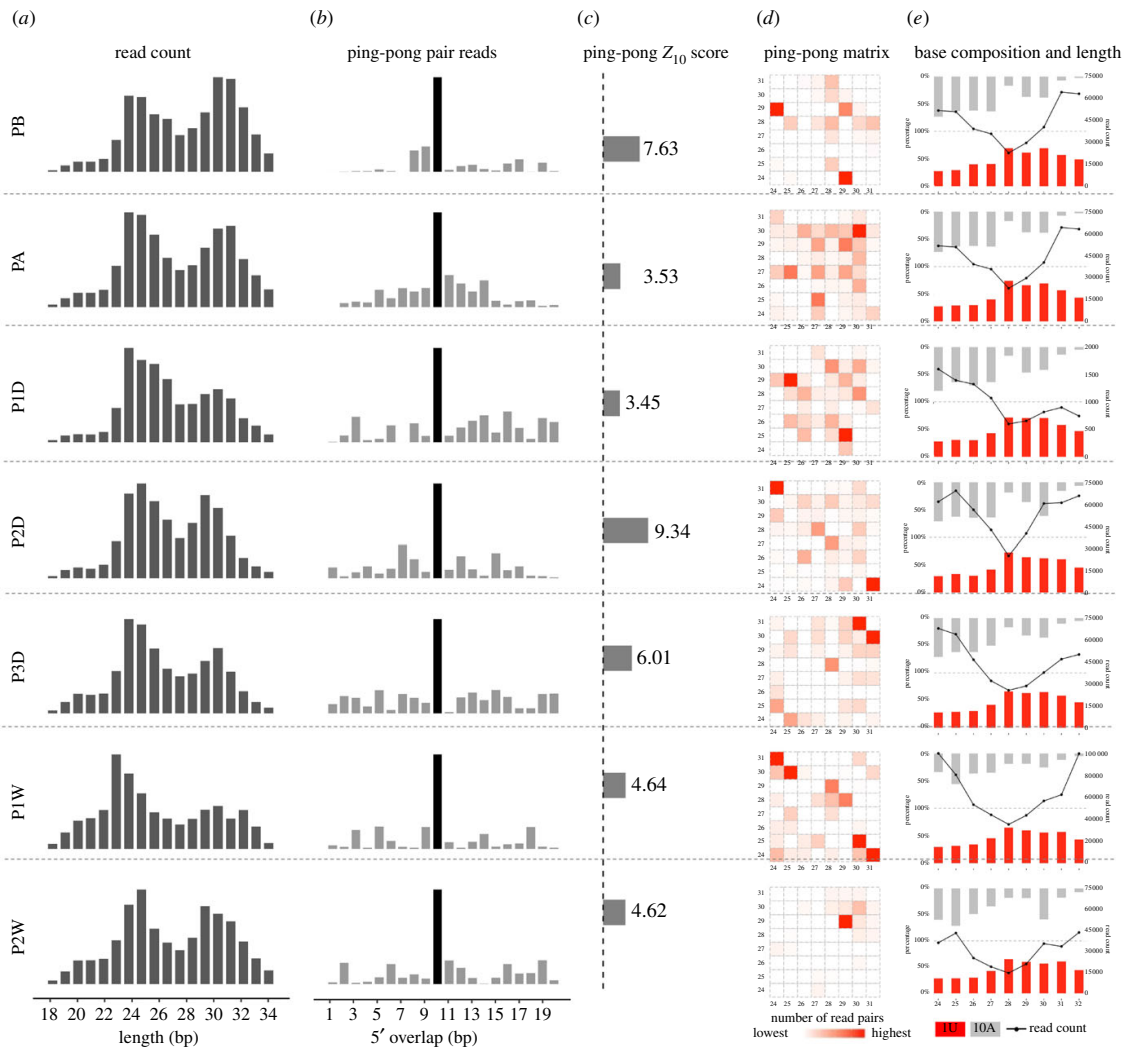
Characteristics of putative PIWI-interacting RNAs (piRNAs) in *Pinctada fucata* exosome. (*a*) Length distribution of filtered reads without known non-coding RNAs; (*b*) ping-pong pair reads of putative piRNAs; (*c*) ping-pong *Z*_10_ score for the enrichment of 10 bp overlaps; (*d*) ping-pong matrices illustrate frequent length combinations of ping-pong pairs (sequences with 10 bp 5′ overlap); (*e*) putative piRNA length distribution and 1U/10A bias for putative piRNA reads.

Except for miRNAs detected in *P. fucata* exosomes, abundant piRNA-sized reads 24/25 nt and 29/31 nt in length were also detected by sRNA-seq (figure 3*a*). All putative reads were merged from all samples and processed for piRNA processing. A total of 277 139 putative unique piRNAs were detected in *P. fucata* exosomes (electronic supplementary material, table S3). The total putative piRNA reads of each library were used for normalization to analyse the expression patterns of piRNAs among different samples according to the RPM method. The expression patterns of piRNAs were not substantially different between the grafting and initial stages, but varied significantly between the initial stages and later stages (electronic supplementary material, figure S1). A significant 10 nt overlap between the sense and antisense strands of putative reads was detected in the small RNAs derived from *P. fucata* exosomes, which is consistent with piRNAs generated by the ping-pong amplification mechanism (figure 3*b*) with significantly enriched ping-pong pairs in each sample (figure 3*c*). Ping-pong matrices also illustrate that the ping-pong pairs combine piRNAs of homogeneous of 28/31 in length and 28/31 and 24/25 nt in length (figure 3*d*). Furthermore, the ping-pong pairs were heavily biased for a 5′ uridine (U) for 28/31 nt piRNAs and stronger bias for an adenine (A) at position 10 for 24/25 nt piRNAs (figure 3*e*). All the evidence reveals the existence of piRNAs in *P. fucata* exosomes.

**Figure 4.**
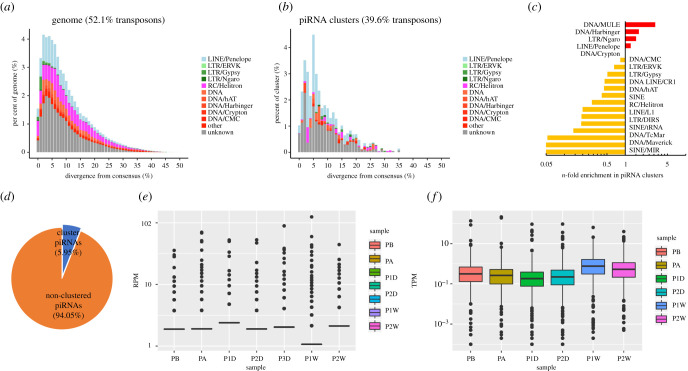
Characteristics of putative PIWI-interacting RNAs (piRNAs) in *Pinctada fucata* exosome. (*a*) Representation of transposons in the genome of *P. fucata*, plotted by divergence (%) from transposon consensus. (*b*) Representation of transposons within piRNA clusters of *P. fucata* exosomes plotted by divergence (%) from transposon consensus; (*c*) prominent transposons that are enriched or depleted in *P. fucata* exosome piRNA clusters; (*d*) fraction of piRNA generated from cluster loci; 5.95% of piRNAs were identified from cluster loci on the genome; (*e*) boxplot of expression patterns of exosomal piRNAs; (*f*) boxplot of expression patterns of transposon elements. TPM, transcripts per kilobase million.

To further parse piRNAs in *P. fucata* exosomes, the putative piRNA reads were merged for clustered locus detection on the genome. A total of 32 piRNA clusters were identified in the genome with a total of 366.94 kb in length. As the background, 539.3 Mb (52.1% of the total genome) of repetitive sequences were annotated in the genome (figure 4*a*). The identified piRNA clusters showed a lower enrichment for transposon sequences than the whole genome, which contained 39.6% transposons (figure 4*b*). Intriguingly, the composition of transposons in piRNA clusters does not at all reflect the transposon landscape of the whole genome. Instead, piRNA clusters were enriched for DNA/Crypton, LTR/Ngaro and LTR/ERVK, showing up to sixfold enrichment in piRNA clusters (figure 4*c*). However, thousands of distinct piRNAs do not cluster on the genome or map to the transposon in *P. fucata* (figure 4*d*). The expression patterns of transposon elements show an opposite tendency of exosome-derived piRNAs, such as a higher expression density of exosome-derived piRNAs in P1D and lower in P1W, as well as a lower expression density of transposon elements in P1D and higher in P1W (figure 4*e,f*).

### 2.5. Target prediction and functional enrichment analysis

**Figure 5.**
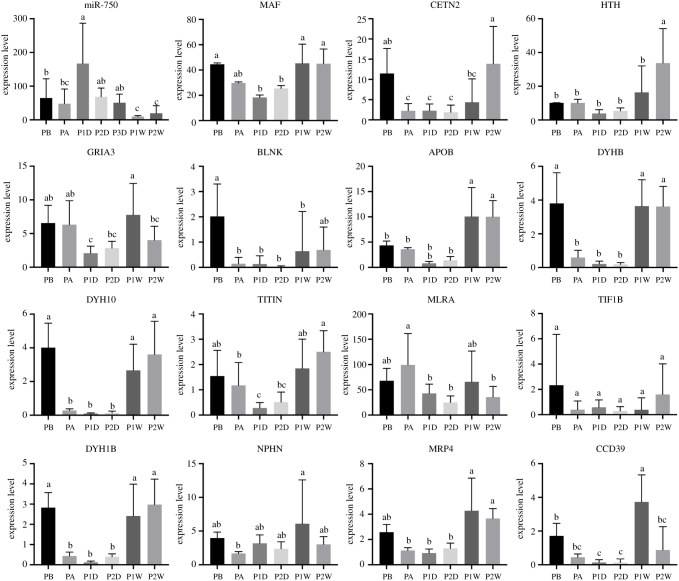
Expression patterns of miR-750 and its predicted target genes. Expression levels are indicated by adjusted transcripts per kilobase million (TPM) values for genes by RNA sequencing, and reads per million (RPM) values for miRNA by small RNA sequencing. Different lowercase letters indicate significant differences in expression levels of miRNAs and predicted target genes (*p* < 0.05).

**Figure 6.**
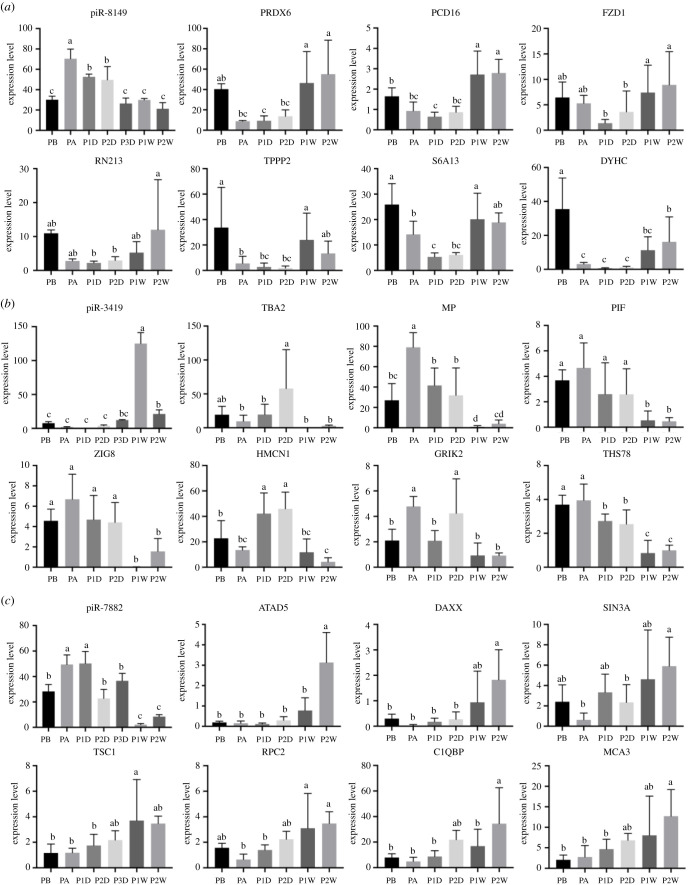
Expression patterns of PIWI-interacting RNAs (piRNAs) and their predicted targets. (*a*) piRNA-8149 and the predicted targets; (*b*) piRNA-3419 and the predicted targets; (*c*) piRNA-7882 and the predicted targets. Expression levels are indicated by adjusted transcripts per kilobase million (TPM) values for genes by RNA sequencing, and reads per million (RPM) values for piRNAs by small RNA sequencing. Different lowercase letters indicate significant differences in expression levels of piRNAs and predicted target genes (*p* < 0.05).

Within the 191 086 unigenes from our previous transcriptome analysis of gene expression performance upon grafting transplantation in *P. fucata*, 8469 and 12 326 unigenes were identified as target genes for all expressed miRNAs and piRNAs, respectively. Enrichment analysis of predicted targets indicated that a vast number of immune-related pathways were significantly (*p* < 0.01) enriched, such as the Wnt signalling pathway (ko04310), cGMP-PKG signalling pathway (ko04022), platelet activation (ko04611), MAPK signalling pathway (ko04010) and various disease-related pathways, such as hepatocellular carcinoma (ko05225) and gastric cancer (ko05226) (table 1; electronic supplementary material, table S4). The Gene Ontology (GO) enrichment analysis also indicated that various immune-related pathways were enriched in the predicted targets of exosomal miRNAs, which is consistent with the results of the Kyoto Encyclopedia of Genes and Genomes (KEGG) enrichment analysis (electronic supplementary material, figure S2). In particular, the predicted targets of miR-750, which were upregulated at the initial stage of grafting transplantation, were enriched in the immune response (electronic supplementary material, figure S3). In addition, the identified piRNAs were also used for target prediction to understand their potential function in gene regulation. A total of 12 326 unigenes were predicted to be targeted by exosomal piRNAs. Enrichment analysis of these predicted targets also indicated that a vast number of immune-related pathways were significantly (*p* < 0.01) enriched, such as the cGMP-PKG signalling pathway (ko04022), calcium signalling pathway (ko04020), Wnt signalling pathway (ko04310), mTOR signalling pathway (ko04150), MAPK signalling pathway (ko04010) and various disease-related pathways (table 1; electronic supplementary material, table S5). Lastly, the expression patterns of miR-750 and three piRNAs (piRNA-8149, piRNA-3419 and piRNA-7882) and their predicted targets were compared. miR-750 was upregulated at the initial stage and downregulated at a later stage upon grafting transplantation, whereas the genes *MAF*, *HTH*, *GRIA3*, *APOB*, *TITIN* and *CCD39,* the miR-750 targets, were downregulated to the initial stage, and upregulated at later stages (figure 5). piR-8149 was immediately upregulated after grafting transplantation and downregulated to initial levels at later stages, whereas the targets of *PRDX6*, *PCD16*, *RN213*, *TPPP2*, *S6A13* and *DYHC* were downregulated after grafting transplantation and upregulated at later stages. piR-3419 was upregulated at a later stage, and the targets of *TBA2*, *MP*, *PIF*, *ZIG8* and *THS78* were downregulated at later stages. piR-7882 was immediately upregulated after grafting transplantation and quickly returned to its initial level and downregulated at a later stage; the targets of *DAXX*, *SIN3A* and *RPC2* showed opposite expression patterns of piR-7882 (figure 6). Among these targets, most are related to the immune response in cellular processes.

**Table 1.** Kyoto Encyclopedia of Genes and Genomes (KEGG) classification of predicted target genes involved in immune-related pathways.

KEGG pathways	pathway ID	no. gene	*p-*value
cGMP-PKG signalling pathway	ko04022	215	5.57 × 10^−18^
Wnt signalling pathway	ko04310	134	1.27 × 10^−14^
calcium signalling pathway	ko04020	215	2.46 × 10^−12^
cAMP signalling pathway	ko04024	226	3.69 × 10^−9^
MAPK signalling pathway—fly	ko04013	135	6.66 × 10^−9^
mTOR signalling pathway	ko04150	167	4.25 × 10^−8^
MAPK signalling pathway	ko04010	231	5.95 × 10^−6^
Hippo signalling pathway	ko04390	149	3.20 × 10^−6^
Fc epsilon RI signalling pathway	ko04664	61	2.09 × 10^−5^
B cell receptor signalling pathway	ko04662	64	7.01 × 10^−5^
TGF-beta signalling pathway	ko04350	57	1.49 × 10^−4^
Epstein–Barr virus infection	ko05169	161	1.54 × 10^−4^
Kaposi sarcoma-associated herpesvirus infection	ko05167	152	2.95 × 10^−4^
Toll-like receptor signalling pathway	ko04620	101	3.80 × 10^−4^
autophagy—animal	ko04140	107	3.87 × 10^−4^
T cell receptor signalling pathway	ko04660	70	6.29 × 10^−4^
AMPK signalling pathway	ko04152	119	6.70 × 10^−4^
human cytomegalovirus infection	ko05163	201	7.58 × 10^−4^
Epstein–Barr virus infection	ko05169	113	8.33 × 10^−4^
human papillomavirus infection	ko05165	232	1.70 × 10^−3^
primary immunodeficiency	ko05340	27	2.28 × 10^−3^
chemokine signalling pathway	ko04062	69	2.54 × 10^−3^
TNF signalling pathway	ko04668	90	2.84 × 10^−3^
Ras signalling pathway	ko04014	186	2.96 × 10^−3^
RIG-I-like receptor signalling pathway	ko04622	51	3.00 × 10^−3^
Th17 cell differentiation	ko04659	51	3.30 × 10^−3^
natural killer cell-mediated cytotoxicity	ko04650	54	3.39 × 10^−3^
autophagy—other eukaryotes	ko04136	37	4.02 × 10^−3^
EGFR tyrosine kinase inhibitor resistance	ko01521	56	4.32 × 10^−3^
RIG-I-like receptor signalling pathway	ko04622	67	8.20 × 10^−3^

## 3. Discussion

Nuclear transfer is often used to produce pearls on a large scale; however, the grafting transplantation process causes pearl oyster stress [33]. Exosomes, a cell-to-cell communication tool, are small membrane-enclosed vesicles actively released by cells into the extracellular environment [44]. The functions of mollusc exosomes are mysterious and diverse, and they are currently known to play an important role in immune response and biomineralization [10–12,28]. In this study, we successfully extracted exosomes from the haemolymph of *P. fucata*, a well-studied mollusc in biomineralization, and further explored the carrying small RNA molecules and their functions during artificial grafting transplantation. TEM showed typical exosome morphology with a spherical shape, and the size of exosomes was analysed using a NanoSight LM10 system with an average size of 170 nm in diameter, which was slightly larger than that of regular exosomes in size, but consistent with extracted exosomes in molluscs of *Crassostrea gigas* and *Hyriopsis cumingii* [10–12].

Exosomes carry cargo molecules that play a pivotal role in cell-to-cell communication, reflecting the physiological or pathological state of donor cells [22,45]. As one of the most important types of small non-coding molecules in exosomes, miRNAs play an important role in intercellular communication by mediating the repression of critical mRNA targets in neighbouring or distant recipient cells [46], while the function of specific miRNA in EVs is still debatable in many animals [47]. Small RNA molecules were isolated and sequenced using sRNA-seq from *P. fucata* exosomes. A total of 38 miRNA species were identified in the examined samples during grafting transplantation, which was fewer than those identified from sRNA-seq from somatic and gonadal tissues and genome-wide prediction [41,48]. Furthermore, the expression levels of most miRNAs were very low in the exosomes. Among these identified miRNAs, miR-67 and miR-750 increased at the initial stage, and miR-87 and novel-1 were upregulated at the later stage of grafting transplantation.

Prediction of miRNA targets is of central importance for a functional understanding of miRNAs in *P. fucata* exosomes. We performed a computational prediction of the targets of exosomal miRNAs based on the assembly unigenes of our previous transcriptome analysis of grafting transplantation in *P. fucata*. Thousands of targets were identified based on physical sequence interactions with these miRNAs. The computational prediction of miRNA targets always yields a large proportion of false positives [49–51]. A complex mRNA–miRNA network was predicted by computational methods in molluscs, which will require a huge amount of experimental work to confirm the predicted targets. However, it does not affect the understanding of the functional landscape of the whole miRNAs in exosomes. In molluscs, the immune response that occurs during the grafting process plays a vital role in response to host oyster survival and regeneration, and immune-related genes are enriched at the initial stage of mantle grafting transplantation [33]. In this study, functional annotations of GO and KEGG pathways also indicated that immune response-related pathways were enriched in the predicted target genes. The target prediction of miR-750 also indicated that this miRNA is related to the immune response. In fact, miR-2, an invertebrate-specific family member, plays a vital role in the immune response [52], and miR-29 is involved in the immune response by regulating neuropeptide Y receptor 2 (Y2R) in *Pinctada martensii* [53]. In addition, several miRNAs, namely miR-750, miR-1175, miR-1990 and miR-1991, were also observed in *P. fucata* exosomes, which were specifically discovered in molluscs by genome-wide prediction [48]. Specifically, miR-1990 showed a mantle-specific expression pattern in *P. fucata* [41], and miR-1991 was specifically expressed in the adductor muscle of *C. gigas* [54] and exosome-derived miR-15b negatively regulated *hcApo* to mediate shell colour formation in *H. cumingii* [11]. All the evidence indicates that exosomes play an important role in various immune responses, growth, development and biomineralization processes in molluscs.

piRNAs are a widespread strategy to effectively suppress transposable element activity to safeguard the genome from detrimental insertion mutagenesis, a common issue in the germline of most animals and higher organisms [55]. Intriguingly, piRNAs have been discovered in the soma of arachnids [56], cnidarians [57,58] and molluscs [41,59,60]. In the present study, abundant exosome-derived piRNAs with canonical characteristics were first discovered in molluscs; these provide important insights into the evolutionary origins and functional understanding of piRNAs in bilaterians. Approximately 10% of unique piRNAs were clustered into 32 piRNA clusters. In line with the transposon element suppressive role of piRNAs, these piRNA clusters show a lower enrichment for transposon elements than the whole genome. Particularly, the expression level of piRNAs was decreased in P1W, whereas the expression levels of transposons increased accordingly. Exosome-derived piRNAs from the haemolymph may be transmitted to the gonadal tissue and act on the germline cells to suppress transposon activity; however, thousands of distinct piRNAs from exosomes do not map to any transposon sequences, indicating a potential additional target and function of piRNAs in *P. fucata*.

Recently, sufficient evidence has supported the involvement of PIWI/piRNA pathways in protection against invading viruses in invertebrates and vertebrates [61,62]. In flatfish, piRNAs were identified from skin mucus exosomes to establish an effective immune response to bacterial infection in *Cynoglossus semilaevis* [63]. Neural stem cell-derived exosomes produce piRNAs that are potentially enriched during the adaptive immunity-like reaction in mice [64]. miRanda, an RNA–RNA interacting prediction tool, was used to predict potential target genes of exosome-derived piRNAs [65]. Tens of thousands of genes were predicted to be targets by exosome-derived piRNAs. Enrichment analysis of these targets implied that exosome-derived piRNAs might also be related to the immune response in *P. fucata* during grafting transplantation.

Despite the extensive studies on molecules and pathways of immune response in molluscs, the repertoire of exosome-derived sncRNAs composing the mollusc immunity remains to be revealed. In this study, pathway analysis based on the GO and KEGG databases was performed to characterize the functional consequences of exosome-derived sncRNAs associated with the immune response upon grafting transplantation in *P. fucata*. Consequently, these immune-related pathways, which have been comprehensively determined in numerous species, were characterized. These pathways included the cGMP-PKG, MAPK, Wnt and mTOR signalling pathways, which are both enriched by miRNA and piRNA targets, suggesting an important role of these pathways in immune response upon grafting transplantation. The mitogen-activated protein kinase (MAPK) plays a conserved role in the regulation of various molecular and physiological processes, including cell differentiation and proliferation, cell growth and death and immune response [66]. A MAPK homologue, *MKK4*, was found to be involved in the response to the nucleus insertion operation, indicating its role in host defence mechanisms, potentially in protecting *P. fucata* from injury caused by grafting or disease [67]. Moreover, the MAPK signalling pathway was also enriched at the early stage of pearl sac and pearl formation via grafting transplantation in *P. fucata* [33]. In conjunction with the activation of the Ras signalling pathway, MAPK activation induces the expression of multiple genes that regulate the inflammatory response [66]. mTOR is an evolutionarily conserved serine/threonine protein kinase from yeast to mammals that senses and integrates a variety of cellular physiological and environmental signals to regulate cell growth [68]. Dephosphorylation of mTOR contributes to induced autophagy responses to contaminants in marine mussels (*Mytilus galloprovincialis*) [69]. In addition, the potential target genes related to immune response were screened based on the expression level of transcriptome analysis and trends of miRNA and piRNAs, which could provide a basis for functional studies of these miRNAs and piRNAs in the future. The findings of small RNAs in exosomes contribute to the expanding knowledge base for miRNA and piRNA analysis in molluscs as well as for elucidating the ancestral state of somatic piRNAs in bilaterians.

## 4. Material and methods

### 4.1. Experimental animal and sample collection

Approximately 2-year-old healthy *P. fucata* pearl oysters were used as donors and recipients for mantle grafting transplantation. Grafting transplantation was performed by a skilled expert at the Mikimoto Pearl Research Institute, Mie Prefecture, Japan. During the grafting transplantation, we collected seven groups of grafting samples: before grafting (PB), just after grafting (PA), 1 day (P1D), 2 days (P2D), 3 days (P3D), 1 week (P1W) and 2 weeks (P2W) post-grafting. The blood lymph fluid was collected from the adductor muscle of five individuals from each group and mixed for exosome isolation. The mixed haemolymph was centrifuged at 2000*g* for 30 min to yield the supernatant and stored at −80°C until use.

### 4.2. Exosome isolation and observation

Exosomes were isolated from the stored supernatant using a Total Exosome Isolation (from serum) Kit (Invitrogen, Carlsbad, CA, USA) following the manufacturer's instructions. The purified exosome solution (10 µl) was crossed with a hydrophilic collodion membrane for 15 min and treated with 1% uranyl acetate for 30 s. The grid was then placed on a TEM sample table for observation and photographed using iTEM. The purified exosome solution was also quantified and sized using the NanoSight LM10 system (Malvern Panalytical, UK) following the manufacturer's instructions.

### 4.3. Exosomal smRNA extraction, library construction and sequencing

Total RNA was extracted from the resuspended exosomes using the Total Exosome RNA and Protein Isolation Kit (Invitrogen) according to the manufacturer's protocol. The quantity and quality of exosomal total RNA were assessed using a Qubit RNA Assay Kit in a Qubit 3.0 Fluorometer (Life Technologies, Carlsbad, CA, USA) and Agilent 2200 TapeStation (Agilent Technologies, Waldbronn, Germany), respectively. Small RNA-seq libraries were constructed using the SMARTer smRNA-Seq Kit for Illumina (Takara, San Jose, CA, USA) following the manufacturer's recommendations. In brief, total RNA was first polyadenylated to provide a priming sequence for an oligo(dT) primer and then the first-strand cDNA molecules were synthesized by adding a second adapter sequence to the 3′ end. Full-length Illumina adapters were subsequently linked during polymerase chain reaction amplification. The cDNA libraries were tested using the High Sensitivity D1000 ScreenTape Kit in Agilent 2200 TapeStation (Agilent Technologies). The clean-up products were purified and size selected using AMPure XP beads (Beckman Coulter, CA, USA) to obtain the appropriate size. The post-size-selection library was also validated by Agilent 2200 TapeStation (Agilent Technologies) with an expected size of 180 bp that combined the size of processed small RNAs plus adapters, followed by sequencing on an Illumina HiSeq 2500 platform with a 50 bp single-end module (Macrogen, Tokyo). Three independent small RNA libraries were constructed and sequenced for each time node sample.

### 4.4. Small RNA sequencing data processing

The raw sequencing data were saved as FASTQ files. We first filtered out the reads with low quality, reads containing a strong poly(A) tail and reads containing poly(N). The 3′ end adapter sequences were trimmed from sequencing reads using fastx_toolkit (fastx_clipper -a AAAAAAAA -c -l 15 -d 0 -Q 33), followed by size filtration with a length range of 15–35 nt. The clean reads were then mapped with the reference genome sequences [70] and Rfam [71] to remove genome unmapped reads and rRNA, tRNA, snRNA and snoRNA sequences using bowtie (v1.2.1, -f -k 3 -v2). The filtered reads were pooled to perform miRNA prediction with miRBase 22.08 using miRDeep2 (v. 2.0.0.8) using default parameters [72]. The identified miRNAs in exosomes were also compared with previously discovered miRNAs in *P. fucata* [41,42] and *C. gigas* in MirGeneDB 2.1 [43]. The miRNA family was annotated according to a previous study [73]. The total miRNA reads of each library were used for normalization to analyse differentially expressed miRNAs among different samples according to the RPM method. A read found more than five times was considered a potential miRNA in *P. fucata* exosomes. The remnant reads that were within 24–32 nt in length and did not produce a match to any known ncRNAs were considered putative piRNAs and subsequent piRNA discovery. PPmeter [59], unitas [74] and proTRAC [75] were used to quantify and compare the amount of ongoing ping-pong amplification, search ping-pong signatures, calculate a *Z*-score for the enrichment of 10 bp overlaps and predict genomic piRNA clusters, respectively. For repeat annotation, we performed a de novo prediction of repetitive elements in the *P. fucata* genome using RepeatMasker (v. 4.0.7) [76] based on a de novo repeat library, constructed using RepeatModeler (v. 1.0.11) [77]. Analysis was conducted with the entire repeat dataset as well as with repeats localized in the predicted piRNA clusters.

### 4.5. Target prediction and enrichment analysis

A gene set of transcriptome analysis from our previous study on gene expression performance during grafting transplantation in *P. fucata* was used for miRNA and piRNA target prediction. The assembly unigenes were aligned NR, Swiss_Prot, COG, GO and KEGG databases using the Triotate pipeline [78]. The 3′UTR sequences were isolated based on the annotation results. Target predictions for miRNAs and piRNAs were performed by miRanda with restricted conditions of a score greater than 150 and a free energy ≤15 kcal mol^−1^ [79]. Enrichment analysis of predicted targets was performed using the OmicShare tools, a free online platform for omics analysis (http://www.omicshare.com/tools).

## 5. Conclusion

In the present study, exosomes were successfully extracted from the haemolymph of *P. fucata* (Mollusca) after transplantation. A vast number of exosome-derived sncRNAs have been identified using sRNA-seq. Intriguingly, in addition to the detection of tens of miRNAs, abundant piRNAs were also discovered in the exosomes. The expression patterns of the miRNAs and piRNAs at the grafting and initial stages were not substantially different, but varied significantly between the initial and later stages. Target prediction and functional analysis indicate that these miRNAs and piRNAs are related to the immune response upon grafting transplantation, whereas piRNAs might also be associated with transposons but are not clear in detail. This work provides the basis for a functional understanding of exosome-derived sncRNAs in molluscs and helps to gain further insight into the PIWI/piRNA pathway function outside of germline cells in invertebrates, which might prove to be applicable to other species in the future.

## Data Availability

All data are presented in the figures and tables or in the electronic supplementary material, figures and tables. The sequencing data are deposited in the DNA Data Bank of Japan (DDBJ) database under accession no. DRA012552. The data are provided in the electronic supplementary material [80].
